# The Colliery Disaster

**Published:** 1896-02-01

**Authors:** 


### The Colliery Disaster.

The terrible colliery explosion in South Wales, by which so many good workers have lost their lives, draws attention again to the grave risks under which some industrial pursuits are carried on, while, as the bright side of a sad catastrophe, it shows how willing brave men are to risk their lives in the attempt to rescue their fellow workmen, however perilous may be the position in which they may be placed; and in this regard we are proud to record the fact that among the first to go down one of the shafts was a medical man, Dr. Morris, who at once volunteered to descend the pit to attend to the injured.

It seems sad that notwithstanding all the advances of science it should still be impossible entirely to prevent the occurrence of these explosions, with all their attendant miseries. Nevertheless, knowledge of the causes of these accidents has of late years greatly increased, and we cannot doubt that very often the means now taken are effectual in preventing explosions which without such measures would certainly have occurred. The influence of varying barometric pressure in setting free the fiery gases which gradually ooze out of the substance of coal is well recognised. What is most to be dreaded in this regard is the occurrence of a fall, even the slightest, after a prolonged period of high barometer. An inspection of the weather chart shows that, after a period of anti-cyclonic weather, our western shores were on Sunday evening caught in the southern edge of a cyclonic depression which was approaching the west of Scotland and during Monday passed away to the north of the Shetland Islands. On Sunday evening a fall of the barometer was noted at the western stations; on Monday it had become general. Early on Monday morning, then, the explosion occurred; and if one considers that a fall of one-fifth of an inch means such an expansion of the gases hidden in the crevices of the coal measures that for every square yard of it over a gallon will escape into the mine, and that every gallon of gas will render a hundred gallons of air explosive, it is not difficult to understand the extreme danger that accompanies such a fall of the barometer after a period of fine weather.

Various investigations, however, many of which have been made by direction of the Home Office, have demonstrated the very great importance of coal dust in extending to vast dimensions explosions which, but for it, might have remained quite local. In consequence of the growing knowledge on this point some increased attention has of late years been paid to keeping mines cleaner than they used to be, and to the prevention of accumulations of this dangerous material; but it is to be feared that no amount of precaution will entirely abolish the risk arising from this cause, for when the cold air is driven into the warm pit it becomes so dry that the whole of the ""face along which it traverses is deprived of moisture to such an extent that the slightest fall of coal provides dust enough to render air explosive, and to lead to a catastrophe such as we have heard of this week. It is curious that the extensive ventilation to which we have so largely trusted for the prevention of the accumulation of explosive gases, and which has been so effective for that purpose, should, by its drying effect, have proved in turn the means of sometimes even increasing the violence of such explosions as do occur. We suppose the next thing to do will be to moisten the air which is driven into the mine; when, probably, the breath of the men will suffer as they did in the cotton sheds in Lancashire. So does the reformer vainly toil like Sisyphus of old, at every step encountering fresh and unthought of obstacles which in turn have to be rolled away.

